# Non-invasive ML methods for diagnosis of congenital heart disease associated with pulmonary arterial hypertension

**DOI:** 10.3389/fphys.2024.1502725

**Published:** 2025-01-03

**Authors:** Yuyang Gao, Pengyue Ma, Jiahua Pan, Hongbo Yang, Tao Guo, Weilian Wang

**Affiliations:** ^1^ Country School of Information Science and Engineering, Yunnan University, Kunming, China; ^2^ Fuwai Yunnan Hospital, Chinese Academy of Medical Sciences, Affiliated Cardiovascular Hospital of Kunming Medical University, Kunming, China

**Keywords:** congenital heart disease associated with pulmonary arterial hypertension, machine learning, segmentation, heart sounds classification, ensemble learning

## Abstract

**Objective:**

Congenital heart disease with pulmonary arterial hypertension (CHD-PAH), caused by CHD, is associated with high clinical mortality. Hence, timely diagnosis is imperative for treatment.

**Approach:**

Two non-invasive diagnosis algorithms of CHD-PAH were put forward in this review, which were direct three-divided and two-stage classification models. Pre-processing in both algorithms focuses on segmentation of heart sounds into discrete cardiac cycles. Both the dual-threshold and Bi-LSTM (Bi-directional Long Short-Term Memory) methods demonstrate efficacy. In the feature extraction phase, the direct three-divided model integrate time-, frequency-, and energy-domain features with deep learning features. While the two-stage classification model sequentially extracts sub-band envelopes and short-time energy of cardiac cycle. In the classification phase, considering the lack of CHD-PAH data, ensemble learning was widely used.

**Main results:**

An accuracy of 88.61% was achieved with direct three-divided model and 90.9% with two-stage classification model.

**Significance:**

By analyzing and discussing these algorithms, future research directions of CHD-PAH assisted diagnosis were discussed. It is hoped that it will provide insight into prediction of CHD-PAH. Thus saving people from death due to untimely assistance.

## 1 Introduction

### 1.1 Formation principle of CHD-PAH

Congenital Heart Disease (CHD) is the result of abnormal development of the heart structure during fetal period (Liu, 2021). The incidence of CHD is related to the region, race, gender, pregnancy environment and other factors, the incidence rate is roughly 6‰ ∼ 8‰ globally, and 2.9‰ ∼ 16‰ in China. Every year, 15 ∼ 200,000 patients are newly added, and Yunnan, as the hardest-hit area of CHD, is about 8 ∼ ‰12‰ (Liyuan, 2022). In 2022, hospitals admitted 1,508,000 inpatients with CHD (Report and Hu, 2023). In congenital heart defects, the pressure and volume of the pulmonary circulation are overloaded by large intracardiac and extracardiac blood flow exchange. Unless intervention is made by cardiac surgery in the early stage, most types of CHD develop with a left-to-right shunt, which leads to excessive pressure in the pulmonary circulation. Continued deterioration will lead to the development of congenital heart disease associated with pulmonary arterial hypertension (CHD-PAH) (Kuwana et al., 2020). Approximately 10% of patients with CHD have PAH, and patients with CHD-PAH account for nearly one-third of adult PAH patients (Ferrero et al., 2024).

Long-term abnormal pulmonary blood flow movement will lead to increased pulmonary vascular pressure and resistance, which may be life-threatening. End-stage PAH patients are typically offered heart-lung transplantation. In the current era, the median survival following heart-lung transplantation, conditional on survival to 1 year post-transplantation, is 12.8 and 8.8 years, respectively. A 32-year analysis of the International Society for Heart and Lung Transplantation (ISHLT) registry reported a 10-year survival rate of 52% in patients with Eisenmenger syndrome, the most extreme form of CHD–PAH (Ferrero et al., 2024), particularly those with atrial or ventricular septal defects. Recent studies of CHD-PAH patients undergoing heart-lung or bilateral lung transplantation in France, including a subset with complex anatomical abnormalities, demonstrated a median survival of 11.2 years and a conditional survival of 14.2 years. Notably, mortality on the waiting list was 34% at 1 year (Jansen et al., 2021). However, a clinical cure can be achieved at the early stage through cardiac surgery. Therefore, early diagnosis of CHD-PAH is of great importance to reduce clinical mortality.

### 1.2 Current diagnostic methods

Invasive right heart catheterization is the gold standard for confirming CHD-PAH (Inampudi et al., 2019). However, this method is not suitable for the screening stage, as it not only technically demanding for the operators, but can also be damaging (Elgendi et al., 2018).

In addition to this, echocardiography can be used for screening (Humbert et al., 2022). Doctors use echocardiographs to scan the blood flow in the patient’s heart to draw an ultrasound picture, which allows them to observe pathological features. This non-invasive test does not cause damage to the human body, but echocardiography is expensive and not widely available in every primary care setting. Therefore, this method cannot be popularized.

Cardiac auscultation is also a common method in screening CHD. As cardiac activity is cyclical, heart sounds are quasi-periodic signals. A single heart sound often contains multiple cardiac cycles. According to the order of appearance, a complete cardiac cycle consists of four parts: S1, systole, S2, and diastole (Das et al., 2020; Chen et al., 2020a). Of these, S2 is caused by the closure of the semilunar valve, containing the aortic component (A2) and the pulmonary artery component (P2) (Sun et al., 2023; Thiyagaraja et al., 2018; Seepana and Vala, 2020). When pulmonary artery pressure increases, the right ventricle requires more force to pump blood into the pulmonary artery, which is accompanied by longer blood injections. This results in the P2 component being enhanced and appearing later than A2 (Aggarwal et al., 2021; Chen et al., 2020b). Therefore, it is feasible to make a supplementary diagnosis of CHD-PAH based on heart sounds. As an aid to diagnosis, auscultation has the advantage of being non-invasive and cheap. However, the number of physicians with auscultation capabilities is limited, and the incomplete information recorded by the human ear may lead to the omission of pathological information (Deng and Han, 2016). Machine learning-based diagnostic algorithms overcome issues like subjectivity in manual auscultation. By digitizing heart sound acquisition and automating diagnosis, they make screening more efficient.

### 1.3 Innovations

The innovation of this paper can be summarized as:

• Current academic researches on heart sound signals are mostly on identifying CHD, with fewer studies on PAH, and even fewer on CHD-PAH. Instead, this review paper systematically summarized the research progress of our research group in the field of computer-aided heart sound detection for CHD-PAH.
• The two algorithms were analyzed in detail in this paper, and possible improvement measures of the two algorithms were listed.
• The future research directions of computer-assisted heart sound detection technology were discussed.

## 2 Literature review

### 2.1 Related work

At present, domestic and foreign researchers mainly use the phonocardiogram (PCG, graphical representation of heart sound signals) to roughly determine whether the signal is CHD or not, which generally includes three steps: preprocessing, feature extraction, and classification. For example, in the preprocessing stage of Xu et al. (2022), segmentation of the cardiac cycle was achieved, which reduces the impact of local noise on the global signal. Subsequently, 84 features were extracted from time and frequency domain. Finally, the classification of CHD was achieved by using Random Forest and Adaboost classifiers. Although the existing studies are not specific to CHD-PAH, they may serve as a cornerstone for related research. However, it is questionable whether the above studies can be directly applied to assisted diagnosis of CHD-PAH.

Since the heart sound frequency in PAH patients is significantly reduced in relative power in the 21–22 Hz (Elgendi et al., 2014). Elgendi et al. (2015) extracted the relative power, entropy and sinusoidal formant energy of the relevant frequency bands in, and finally used a linear discriminator to analyze whether PAH was affected. However, most PAH patients develop from the aggravation of CHD patients, so early detection of PAH in CHD patients is particularly important.

### 2.2 Datasets

Two heart sound datasets were used in the algorithms reviewed in this paper.

• The PhysioNet/CinC 2016 public dataset (publicly available dataset) (Liu et al., 2016): The dataset was divided into 8 subsets (a ∼ i), where subsets a ∼ f are public. A total of 3,240 heart sound recordings were included. These heart sound recordings can be divided into two categories: normal and abnormal, with a ratio of about 4:1. Abnormal signal were not labeled with a specific disease.
• The heart sound dataset established by our research group and the Fuwai Cardiovascular Hospital of Yunnan Province (self-constructed dataset): The data were collected using two generations of heart sound acquisition devices developed by our research group. The first-generation device can collect electrocardiogram (ECG) and PCG synchronously. ECG records the electrical activity of the heart using electrodes placed on the skin. It provides information about the heart’s rhythm, electrical conduction, and potential abnormalities such as arrhythmias or myocardial infarction. PCG records the mechanical activity of the heart by capturing heart sounds using a microphone or specialized sensor. It is primarily used to detect S1, S2 and murmurs, providing insights into heart valve functionality and blood flow. The second-generation device only collected heart sounds from five cardiac auscultation zones of an individual. A total of 54,650 normal and abnormal heart sounds were recorded, and the age range of the subjects was limited to 6 months to 18 years. All abnormal heart sounds were labeled with hospital-confirmed specific cases.

### 2.3 Direct three-divided model

**FIGURE 1:**
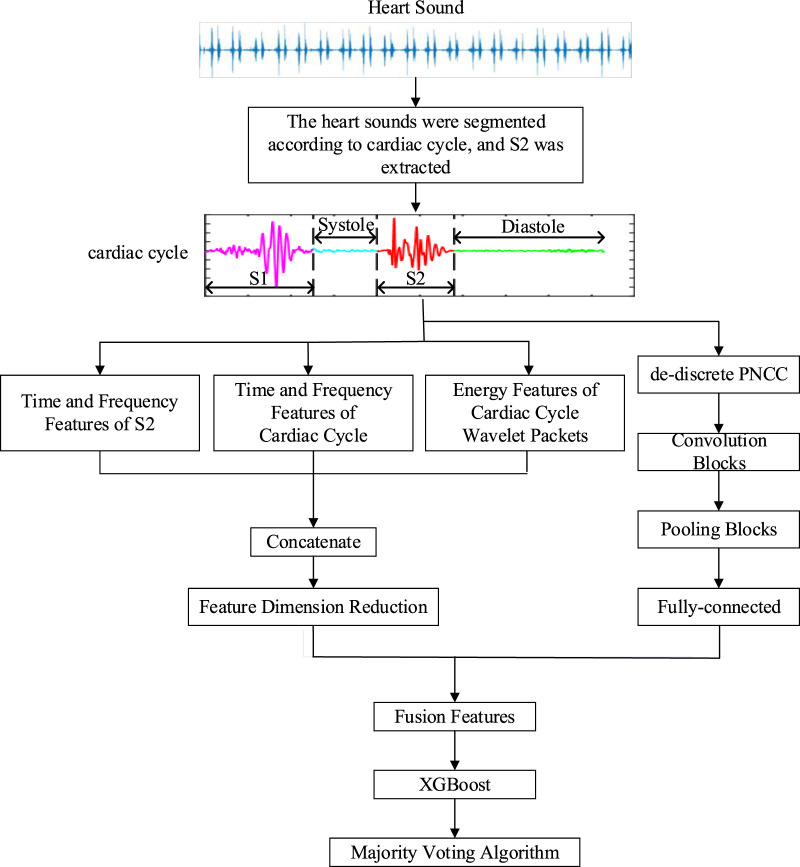
The general framework of the direct three-divided model.

Ge et al. (2023) proposed a direct three-divided model, in which heart sound can be classified into three categories: normal, CHD, or CHD-PAH. Its methodology was described in more detail in Ma et al. (2023). The general framework of the algorithm is shown in Figure 1.

#### 2.3.1 Pre-processing

In the pre-processing phase, accurate segmentation of heart sounds is necessary since the main pathologic features of CHD-PAH patients are concentrated in the S2 component. In segmentation, each state of the cardiac cycle is determined by localizing the S1 and S2 onset positions (Moukadem et al., 2013). However, since heart sound signals are accompanied by murmurs such as lung sounds, breath sounds, and environmental noise, making accurate segmentation of heart sounds a challenging problem.

According to Ge et al. (2023), Ma et al. (2023), the portion of the heart sound signal below 1,000 Hz contains all the valid pathologic information about PAH, so the heart sound signal was downsampled from a sampling frequency of 5,000 Hz–2,500 Hz. By reducing the number of sample points, the computing time was greatly reduced without losing any useful information. Then the threshold detection was used to locate S1 and S2. Finally, the cardiac cycles and S2 components were saved.

The specific segmentation process is as follows:

A Hamming window with a window length of 0.1s and no overlap was used to divide the heart sounds into frames. Next, the short-time energy $E_{i}$ and spectral spread $S_{i}$ of each frame were calculated as Equations 1, 2. Let $x_{i}\left(n\right),n=1\cdots N$ represent the *i*th frame of heart sounds with length N, $f_{i}$ denote its frequency, and $s_{i}$ the spectral value of the *i*th frame. The boundary values $b_{1}$ and $b_{2}$ are used to compute the spectral spread, while $\mu_{1}$ refers to the spectral centroid.

**FIGURE 2:**
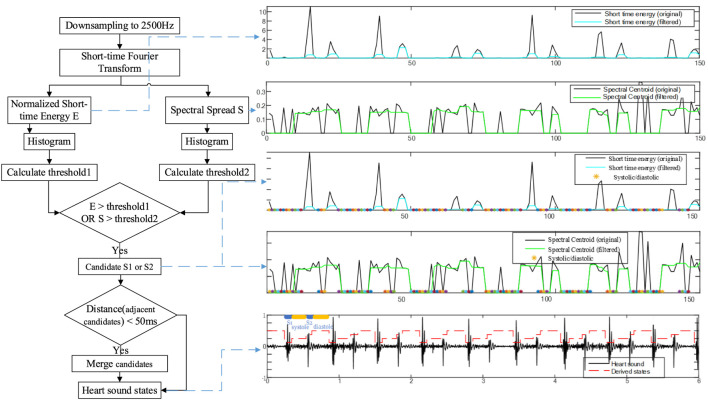
Step-wise procedure of dual-threshold segmentation.

Since S1 and S2 exhibit significantly higher energy than systolic and diastolic, histograms of short-time energy and spectral spread were created separately. Based on these histograms, dynamic thresholds were computed using Equation 3. Where $M_{1}$ and $M_{2}$ represent the positions of the first and second largest peaks in the histogram, respectively, and W is a fixed constant. When the short-time energy or spectral spread exceeded their corresponding thresholds, candidate S1 or S2 were identified. If the distance between adjacent candidates was less than a predefined merge distance (set to 50 ms), the two candidates were merged. Finally, the remaining candidate points were mapped to the original signal. Based on the principle that the diastolic has the longest duration of the four states in the cardiac cycle (Luisada et al., 1949), the starting object of the longest segment was labeled as S2 and the ending object as S1. Then the remaining points were labeled in turn. The flow of the segmentation algorithm is shown in Figure 2. However, this segmentation method relies on specific features and its robustness has yet to be verified.

$$E_{i}=\frac{1}{N}\sum_{n=1}^{N}|x_{i}\left(n\right){|}^{2}$$ (1)

$$S_{i}=\sqrt{\frac{\sum_{i=b_{1}}^{b_{2}}{\left(f_{i}-\mu_{1}\right)}^{2}s_{i}}{\sum_{i=b_{1}}^{b_{2}}s_{i}}}$$ (2)

$$T=\frac{W\cdot M_{1}+M_{2}}{W+1}$$ (3)

#### 2.3.2 Feature extraction

When training data is limited, the performance and computational efficiency of the model may be improved by using traditional feature extraction. It does so by analyzing the data and condensing the most important features. In the feature extraction stage, a new fusion feature that can make full use of both traditional methods and deep learning techniques has been proposed.

For time-domain features, the intensity, amplitude, duration, and time interval are important factors in the judgment of auscultation. In addition, the phase corresponding to the maximum and second largest value, and the phase difference between them of the cardiac cycle and S2 component were introduced as supplementary features to characterize the pathology of CHD-PAH. A total of nine features were selected.

In the frequency-domain, features could be extracted based on the increasing dominant frequency of P2 in CHD-PAH patients. Firstly, the Hamming window was applied to frame the signal. Peaks were found in the power spectral density of the signal in each frame to obtain the dominant frequency. Thus a series of features were extracted based on the dominant frequency of the cardiac cycle and S2.

Different types of heart disease produce murmurs at different periods of the cardiac cycle, while wavelet packets can be well used for time-frequency localization analysis. Therefore, for heart sound signal with a down-sampling frequency of 2,500 Hz, wavelet packet decomposition (Kevric and Subasi, 2017) was used to divide the signal into four frequency bands: 0–156 Hz, 156–312 Hz, 312–625 Hz and 625–1,250 Hz. Thus, the energy features of cardiac cycle wavelet packets were extracted.

In order to reduce the increase in computation caused by a large number of features, the above three types of features were filtered. Features with variances less than 0.05 were filtered. Then XGBoost was constructed to rank the importance of each feature, retaining the most important 148 features. After filtering, time-domain features, frequency-domain features, energy features, and descriptions of them are shown in Table 1, as described in Ge et al. (2023), Ma et al. (2023).

**TABLE 1:** Feature variable list.

Item	Textual description	Mathematical description	Number of features
$i_{cc},i_{s2}$ (Maknickas and Maknickas, 2017)	Intensity of cardiac cycle and S2	$i=\sum_{i=1}^{n}{\left(x_{k}\left(i\right)\right)}^{2}$	2
$I_{ratio}$ (Ge et al., 2023; Ma et al., 2023)	The ratio of intensity between S2 and cardiac cycle	${I_{ratio}=i}_{s2}/i_{ccy}$	1
$l_{cc},l_{s2}$ (Ge et al., 2023; Ma et al., 2023)	Interval of cardiac cycle and S2	—	2
$L_{ratio}$ (Ge et al., 2023; Ma et al., 2023)	The ratio of interval between S2 and cardiac cycle	${L_{ratio}=l}_{s2}/l_{ccy}$	1
$M_{s2}$ (Ge et al., 2023; Ma et al., 2023)	The maximum value of S2	—	1
${ave}_{cc}$ (Ge et al., 2023; Ma et al., 2023)	Mean amplitude of cardiac cycle	—	1
${M2}_{s2}$ (Ge et al., 2023; Ma et al., 2023)	The second largest value of S2	—	1
${pos}_{2}$ (Ge et al., 2023; Ma et al., 2023)	The phase difference between the maximum and second maximum of S2	—	1
${mf}_{cc},{mf}_{s2}$ (Ge et al., 2023; Ma et al., 2023)	median frequency of cardiac cycle and S2	—	2
${iqr}_{cc},{iqr}_{s2}$ (Ge et al., 2023; Ma et al., 2023)	Inter quantile range of cardiac cycle and S2	—	2
${sk}_{cc},{sk}_{s2}$ (Nogueira et al., 2019)	Skewness of cardiac cycle and S2	$sk=E\left[{\left(\frac{X-\mu }{\sigma }\right)}^{3}\right]$	2
${ku}_{cc},{ku}_{s2}$ (Nogueira et al., 2019)	Kurtosis of cardiac cycle and S2	$ku=E\left[{\left(\frac{X-\mu }{\sigma }\right)}^{4}\right]$	2
${es}_{cc},{es}_{s2}$ (Ge et al., 2023; Ma et al., 2023)	Spectral entropy of cardiac cycle and S2	—	2
${sfm}_{cc},{sfm}_{s2}$ (Chen et al., 2019)	Spectral flatness of cardiac cycle and S2	$sfm=\frac{{\left[\prod_{k=1}^{K}\left|es\left(K\right)\right|\right]}^{\frac{1}{K}}}{\frac{1}{K}\sum_{k=1}^{K}\left|es\left(k\right)\right|}$	2
${mode}_{cc},{mode}_{s2}$ (Ge et al., 2023; Ma et al., 2023)	Mode frequency of cardiac cycle and S2	—	2
${aff}_{cc},{aff}_{s2}$ (Ge et al., 2023; Ma et al., 2023)	Average fundamental frequency	—	2
${minff}_{cc},{minff}_{s2}$ (Ge et al., 2023; Ma et al., 2023)	Minimum fundamental frequency	—	2
${maxff}_{cc},{maxff}_{s2}$ (Ge et al., 2023; Ma et al., 2023)	Maximum fundamental frequency	—	2
${dd}_{cc},{dd}_{s2}$ (Ge et al., 2023; Ma et al., 2023)	Dominant frequency range	—	2
${sp}_{cc},{sp}_{s2}$ (Ge et al., 2023; Ma et al., 2023)	Spectrum	—	40
$a_{cc},a_{s2}$ (Ge et al., 2023; Ma et al., 2023)	Sum of amplitudes	—	40
${sr}_{cc},{sr}_{s2}$ (Ge et al., 2023; Ma et al., 2023)	Spectral Roll-off	—	32
${wpt1}_{cc}$ (Ge et al., 2023; Ma et al., 2023)	Energy features in 0–156 Hz	—	1
${wpt2}_{cc}$ (Ge et al., 2023; Ma et al., 2023)	Energy features in 156–312 Hz	—	1
${wpt3}_{cc}{iqr}_{s2}$ (Ge et al., 2023; Ma et al., 2023)	Energy features in 312–625 Hz	—	1
${wpt4}_{cc}{iqr}_{s2}$ (Ge et al., 2023; Ma et al., 2023)	Energy features in 625–1,250 Hz	—	1
**Total number**			**148**

The bold values indicate the total number of features.

**FIGURE 3:**
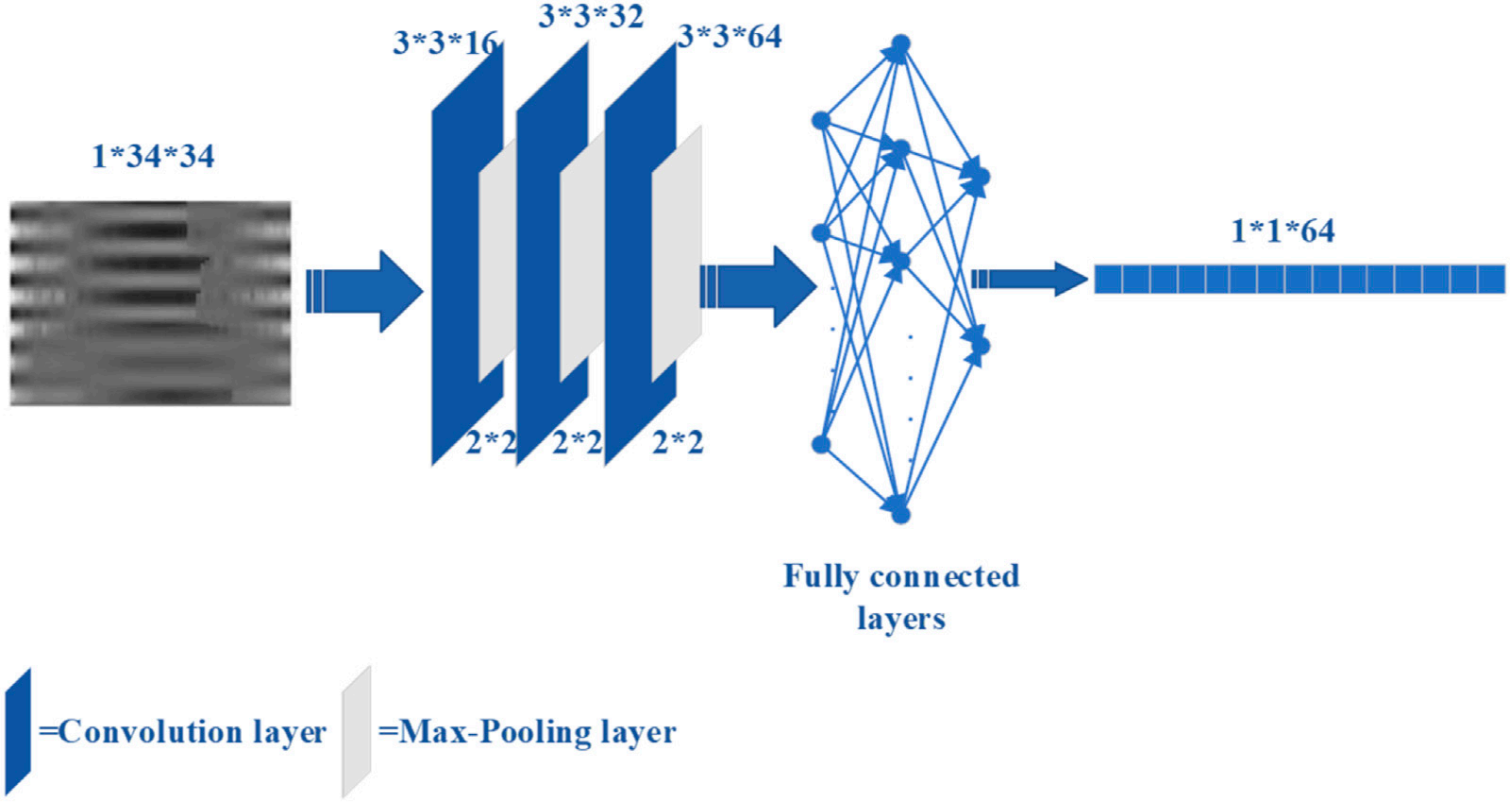
The CNN structure for extracting deep features.

However, traditional feature extraction lacks the ability to extract high-level features from raw data, which is available to deep learning. For the case of insufficient data, the use of deep learning to classify may result in poor performance on the test set. Therefore, convolutional neural network (CNN) (LeCun et al., 1989) was used as an automatic feature extractor to extract deep learning features from the de-discrete power-normalized cepstral coefficients (PNCC) (Kim and Stern, 2016). The structure of the CNN used in Ge et al. (2023), Ma et al. (2023) is shown in Figure 3.

Ultimately, the 212 features were fused, which includes 148 traditional features, and 64 deep-learning features.

However, these features were simply stacked. Although this method is simple, it may lead to some important features being masked or ignored. Other feature fusion methods can be considered, such as introducing an attentional mechanism. This involves learning a set of weights to prioritize important features to increase their impact.

#### 2.3.3 Classification

In the classification phase, XGBoost (Chen and Guestrin, 2016) was used as a classifier since CHD-PAH samples were small but valid pathological information are included in its features. XGBoost reduces the risk of overfitting by adding a regular term to the objective function that controls the complexity of the model.

A 20-s heart sound signal may include 20 to 33 cardiac cycles. The inputs of the XGBoost were features from a single cardiac cycle, so multiple results may be generated for the same recording due to noise interference. Therefore, the majority voting algorithm (Kui et al., 2021) was used to combine the classification results. That is, the classification result with the highest frequency in all cardiac cycles of the sample is considered to be the final classification result of the recording. This reduced the impact of misjudgement of individual cardiac cycles, so the classification accuracy was improved.

### 2.4 Two-stage classification model

**FIGURE 4:**
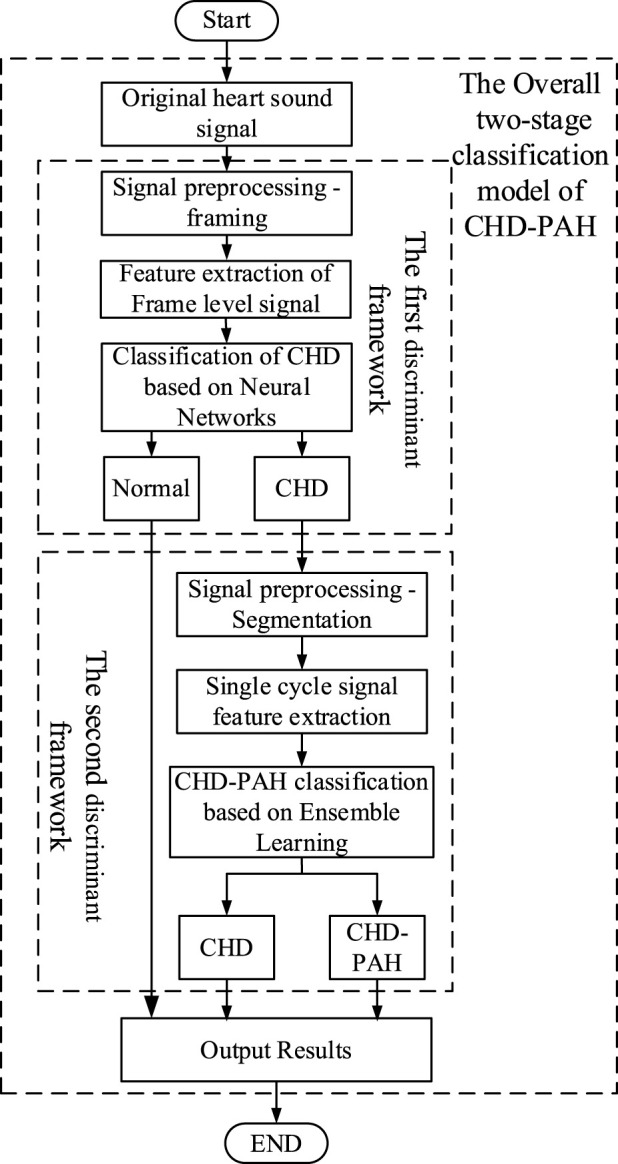
The general framework of the two-stage classification model.

A two-stage classification model was put forward in (Wang, 2022), in which binary classification of normal and abnormal was performed firstly, then the abnormal heart sounds were classified as CHD or CHD-PAH. The overall framework is shown in Figure 4.

#### 2.4.1 The first stage classification model

The first stage classification model realizes the classification of normal and pathological heart sounds. According to Wang (2022), framing was performed in the pre-processing phase, with the aim of increasing the amount of data for training. A Tukey window with a length of 2s (to ensure that at least one full cardiac cycle was present) and an overlap of 1s was used for framing.

Time-frequency domain features can provide a more comprehensive description for signal analysis by simultaneously describing information about changes of the signal in both time and frequency dimensions. A novel time-frequency analysis method was proposed, i.e., constructing time-frequency features by stacking sub-band signal envelopes. The method is computationally simple and effective, which is expected to be used in practical clinical applications. The specific feature extraction process is as follows:

A Gammatone filter set Qi (2013) was used to filter and decompose the framed heart sound signals. The sub-band envelope was computed from the decomposed sub-band signal by means of the Hilbert transform. Average down-sampling of sub-band envelopes was performed to reduce computing time. The logarithmic operation of the sub-band envelope after down-sampling reduced the correlation between the data and compressed the data to make it smoother and easier to calculate. Finally, the sub-band envelopes were transposed and stacked horizontally into a two-dimensional matrix. After centralization and normalization, the sub-band envelope feature map was obtained.

Finally, a shallow CNN was chosen for classification because of the abundant data.

#### 2.4.2 The second stage classification model

The second stage classification model is aimed at the recognition of CHD and CHD-PAH, both of which are pathological signals with weak differences that make classification difficult. Therefore, accurate segmentation of heart sound signals by cardiac cycle was necessary. Signal envelopes are often used in heart sound segmentation. For example, in Xu et al. (2023), the Viola integral envelope of the band-pass filtered signal was extracted and its low amplitude peaks were highlighted with Shannon energy. The mean values of the upper and lower envelopes were used as dynamic thresholds to initially determine the S1 and S2 positions. Aiming at the inevitable large number of omissions and misdetections in the preliminary detection, K-means clustering classifies the distances between the peak points, and removes error points by combining Haar wavelet transform, resulting in the realization of segmentation. In Jamal et al. (2021), a method based on mutation and peak points of signal envelopment have been proposed. However, this method is unable to deal with the interference of murmurs in abnormal heart sounds.

In Wang (2022), a Bi-LSTM network (Song et al., 2023) was built for heart sound segmentation, with envelope as the input to the network. By comparing the results with those obtained by autocorrelation, combined with the intrinsic state transition rules of heart sounds, the segmentation results were further refined.

**FIGURE 5:**
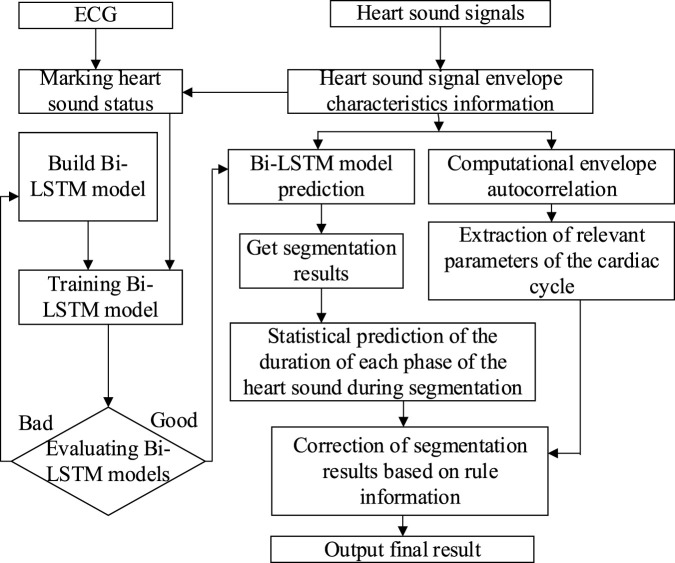
The flow of the Bi-LSTM combined with state-constrained segmentation algorithm.

As is shown in Figure 5, the specific process of the heart sound segmentation algorithm based on Bi-LSTM with state constraints is as follows:

1. There are too many redundant components in the original heart sound, which will directly affect the final heart sound segmentation results. The envelope can reduce the interference of noise, so envelopes of the heart sound signal were extracted, namely the homomorphic envelope (Kamson et al., 2019) (The signal can be regarded as the multiplication of the slowly varying component and the vibrating component. After the logarithmic transformation, the unwanted high-frequency components can be removed using low-pass filtering.), normalized Shannon energy envelope (Choi and Jiang, 2008) (characterize the energy distribution in a signal), and PSD (Power Spectral Density) envelope (Rahman et al., 2021) (The frequency components of S1 and S2 in the heart sound signal are mostly distributed below 150 Hz and concentrated around the 50 Hz frequency (Sharma, 2015). Therefore, the average power spectrum between 40 and 60 Hz is used to form the PSD envelope.).
2. Since the R-peak and T-wave endings in the ECG correspond to S1 and S2 in the PCG respectively, the synchronously collected ECG was used to accurately mark each state of cardiac cycles in the PCG envelope signal (Schmidt et al., 2010; Springer et al., 2015; Renna et al., 2019).
3. The Bi-LSTM network was built to establish bi-directional connections at each time step, and the extracted envelope morphological features were used for training. After the initial segmentation is completed, the duration of each state was counted.
4. Autocorrelation was calculated using the extracted envelopes to obtain the duration of the cardiac cycle (Schmidt et al., 2010). The autocorrelation analysis was performed on the normalized Shannon energy envelope of the signal to eliminate some noise-induced errors. The length of the cardiac cycle can be determined from the origin to the first peak point between 500 and 2,000 ms, while the length of the systole was identified as starting from the origin to the highest point between 200 ms and half the length of the cardiac cycle (Yuenyong et al., 2011).
5. According to the comparison between the statistical duration of the Bi-LSTM output and the duration calculated by the autocorrelation, whether the Bi-LSTM output needs to be corrected can be determined. If corrections are needed, the median duration of each state and the mean duration of the entire cardiac cycle should be calculated. Then, starting from the position of the last correct cardiac cycle marker, the state length was taken as the median of the four states, and was filled in the order of S1, systolic, S2 and diastolic.

Based on the heart sounds of CHD-PAH are characterized by long split tone intervals of S2 and hyperactivity of P2 (Chen et al., 1996; Cobra et al., 2016), short-time energy was added, which is commonly used to calculate the energy emitted by the signal at a given time. By combining the two, not only the envelope information was highlighted, but also the signal energy fluctuation could be shown.

**FIGURE 6:**
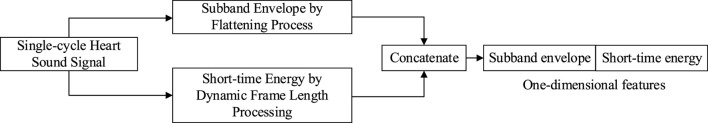
Feature extraction mechanism of single-cycle heart sound signal.

Since sub-band envelope was two-dimensional while short-time energy was one-dimensional, it needs to be flattened before fusion. Specifically, the two-dimensional sub-band envelope features are flattened row-wise. For example, a feature map of size 32 × 16 is flattened into a one-dimensional vector of size 1 × 512. Then, the one-dimensional short-time energy feature, which has a length of nFrame (where nFrame is determined by the signal length), is concatenated to the flattened sub-band envelope vector, completing the feature fusion. For instance, with a sub-band envelope feature size of 32 × 16, the fused feature vector will have a size of 1 × (512 + nFrame). The flow of feature extraction is shown in Figure 6.

In order to obtain the optimal model, three classical machine learning methods, K-Nearest Neighbor (KNN) (Cover and Hart, 1967) (a distance-based classification method, whose basic principle is to search the K training samples closest to the given test point, and the predicted category is the same as the category to which most of the neighboring points belong), Random Forests (RF) (Breiman, 2001) (a classification algorithm based on decision trees. Firstly, n training subsets are obtained to train n decision trees by randomly extracting from the complete training set. The final result is obtained by summarizing all the decision trees.), and support vector machines (SVM) (Cortes and Vapnik, 1995) (SVM tries to find the maximum interval boundaries between different classes and uses the decision boundaries to classify or regress new sample.) were used to compensate for each other, resulting in a reduction of the overall error and an increase in accuracy. After the features had been passed through the three learners, a soft voting method (Kurian and Jyothi, 2023) (using the average output probability of each learner for each category as the final decision criterion) was used to combine the outputs of the three learners.

After the training of the above two classification models was completed, they were concatenated. That is, part of the outputs of the first stage were used as the inputs of the second stage. Finally, the signals were classified into three categories: normal, CHD and CHD-PAH.

## 3 Results

### 3.1 Segmentation results

According to Wang (2022), in the heart sound segmentation experiment, 542 synchronized ECG and PCG were collected from publicly available dataset and self-constructed dataset, including 204 normal data and 338 abnormal signals. These signals were divided into training and test sets according to 8:2.

In order to evaluate the ability of the segmentation algorithm, the segmentation sensitivity (symbolized as ${Se}_{f}$ ), the positive detection rate (symbolized as $P_{+}$ ), and the F1 score (symbolized as $F_{f1}$ ) were used.

The expressions of these evaluation indicators are shown in Equations 4–6. Where, TP (true positive) and TN (true negative) are the number of correctly categorized positive and negative classes, respectively. FP (false positive) is the number of negative classes predicted to be positive and FN (false negative) is the number of positive classes predicted to be negative.

$$Se_{f}=TP/\left(TP+TN+FP+FN\right)$$ (4)

$$P_{+}=TP/\left(TP+FP\right)$$ (5)

$$F_{f1}=\left(2\times Se_{f}\times P_{+}\right)/\left(Se_{f}+P_{+}\right)$$ (6)

In order to further validate the effectiveness of the algorithm, the following segmentation methods were used as comparison groups: DHMM (Kui et al., 2021) (Duration-dependent Hidden Markov Model), LSTM, and Bi-LSTM. The performance comparison of the heart sound segmentation algorithm proposed in Wang (2022) with the commonly used algorithms is shown in Table 2. Among them, DHMM-based heart sound segmentation is commonly used nowadays. The HMM-based method is used to infer the reasonable state sequence by the relationship between the observed sequence and the hidden state sequence. However, there is a limitation that the probability of heart sound transferring to the next state is independent of the current state duration. In DHMM, the state durations were modeled by a Gaussian Mixture Model (GMM) (Rasmussen, 1999). By adding state durations to HMM, it is possible to infer a more reasonable state sequence. However, the Bi-LSTM is better than DHMM in enhancing the information connection between the preceding and following states, so the overall performance is better. A single-layer Bi-LSTM consists of 2 LSTMs (Long Short-Term Memory), one for processing the sequence forwards and one for processing it backwards, so it has better performance in identifying heart sound states than LSTM.

**TABLE 2:** Performance of each heart sound segmentation algorith (mean ± standard deviation).

Heart sound segmentation algorithm	${Se}_{f}\left(\%\right)$		$P_{+}\left(\%\right)$		$F_{f1}\left(\%\right)$	
	Publicly available dataset	Self-constructed dataset	Publicly available dataset	Self-constructed dataset	Publicly available dataset	Self-constructed dataset
DHMM	90.98 ± 1.89	91.20 ± 0.99	94.30 ± 1.44	92.92 ± 0.70	92.62 ± 1.63	92.05 ± 0.77
LSTM	89.75 ± 1.77	86.46 ± 2.97	93.60 ± 1.27	90.59 ± 1.16	91.63 ± 1.26	88.44 ± 1.57
Bi-LSTM	95.84 ± 0.58	93.14 ± 1.79	96.54 ± 0.65	93.26 ± 0.72	96.19 ± 0.48	92.98 ± 0.93
**Bi-LSTM + constraint algorithm**	**96.63 ± 0.47**	**93.54 ± 1.55**	**96.29 ± 0.69**	**93.17 ± 0.35**	**96.46 ± 0.57**	**93.28 ± 0.76**

The bold rows highlight the performance of the segmentation algorithm used in Wang (2022).

When constructing the Bi-LSTM, the number of input layer units is set to 50, and the whole network has 4 hidden layers, each with 100, 200, 100 and 50 units, respectively. Finally, a dense layer with 4 neurons is connected to the network output. The optimizer was chosen as Adam with an initial learning rate of 0.001.

However, overfitting was observed during training, as evidenced by the high accuracy achieved on the training data, while the validation accuracy showed little improvement or even decreased over epochs. This suggested that the model was not generalizing well to unseen data. To address this issue, several mitigation strategies were implemented. Firstly, dropout layers with a random inactivation rate of 0.2 is added after the second and third hidden layers. The addition of dropout layers helps prevent the model from relying too heavily on any specific neuron, encouraging the network to learn more robust features. Secondly, the robustness of the model was further evaluated using a five-fold cross-validation method. In this process, the two datasets used in the heart sound segmentation experiments were randomly and unrepeatedly sampled five times. Each time, four subsets were randomly selected for training, and the remaining one was used as the test, which was repeated five times. This approach helps ensure that the model is exposed to different subsets of the data, providing a more reliable estimate of performance and reducing the likelihood of overfitting.

### 3.2 Classification results

In Ge et al. (2023); Ma et al. (2023), a total of 483 symmetric heart sound signals from self-constructed dataset were used, that is, 161 each of normal, CHD, and CHD-PAH, being randomly divided into 55% training set, 20% validation set, and 25% test set. The optimal XGBoost model parameters, determined through experimentation, are as follows: 600 trees, a tree depth of 7 layers, a minimum leaf node weight of 1, L1 regularization coefficient of 1, L2 regularization coefficient of 3, and a learning rate of 0.01. With these settings, from the classification results shown in Table 3, the model achieves an accuracy of 88.61% on the triple-classification (normal\CHD\CHD-PAH) after majority voting.

**TABLE 3:** The classification results of direct three-divided model.

Type	Precision	Recall	Accuracy
Normal	0.99	0.9268	0.8861
CHD-PAH	0.8571	0.8780	
CHD	0.8536	0.8536	

Precision (that is, the $P_{+}$ mentioned above), recall, and accuracy were used to evaluate the algorithm’s performance. The calculated expressions are shown in Equations 7, 8.

$$Recall=TP/\left(TP+FN\right)$$ (7)

$$Accuracy=\left(TP+TN\right)/\left(TP+TN+FP+FN\right)$$ (8)

In Wang (2022), for the first stage classification model, all 3,240 recordings from publicly available dataset and 5,000 symmetric normal-CHD recordings from self-constructed dataset were used. These recordings were divided into training set, validation set and test set at the ratio of 0.65, 0.15 and 0.2. For the second stage classification model, 1,260 symmetric CHD and CHD-PAH recordings from self-constructed dataset were used. Of these, 1,010 cases were used for the training set and 250 cases for the testing set.

As shown in Table 4, under 725 symmetric data, the classification accuracy of the overall framework is 90.9%. While under asymmetric data samples, that is, the ratio of normal, CHD, and CHD-PAH was 7:2:1 for 600 cases, the overall recognition accuracy was 93.3%. Moreover, the average test time for a recording was 13.3s, ensuring real-time performance.

**TABLE 4:** The classification results of two-stage classification model (dataset A: publicly available dataset, dataset B: self-constructed dataset).

Model	Precision		Recall		Accuracy		$F_{f1}$	
First stage classification model	dataset A	dataset B	dataset A	dataset B	dataset A	dataset B	dataset A	dataset B
	0.92	0.944	0.96	0.96	0.94	0.952	0.94	0.951
Second stage classification model	0.92		0.944		0.932		0.931	
Two-stage classification model	—		—		Symmetric data	Asymmetric data	—	
					0.909	0.933		

Experimental results demonstrate that the classification performance is optimal when the number of time series slices is set to 32 and the number of Gammatone filters is set to 16, resulting in a subband envelope feature size of 32 × 16. Through grid search tuning, the best hyperparameters for the three individual learners—KNN, RF, and SVM—are as follows: for KNN, the optimal K value is 5; for RF, the decision tree type is ID3, the number of trees is 86, and the tree depth is 45; for SVM, the RBF kernel is used with γ set to 0.1 and the penalty coefficient C set to 0.6.

## 4 Discussion

### 4.1 Analysis of relevant references and possible improvement measures

1. Since the features are based on cardiac cycle and S2, the precision of segmentation will greatly affect the final classification results. Segmentation validity can be verified by comparing the segmentation results with the precise localization obtained from synchronized collected ECG signals or other precise segmentation method. The effectiveness of segmentation can also be verified by comparing the effect of the use of segmentation algorithms on the final classification results through ablation experiments.
2. In the preprocessing phase of the second stage classification model, the performance of the segmentation model greatly depends on the Bi-LSTM network. In the subsequent study, the algorithm can be further optimized by adding the heart sound state constraint rules into the Bi-LSTM network. So that it has the ability to restrict the state transfer.
3. In the classification phase of the second stage classification model, when heterogeneously integrating the three learners, the learning method can be considered instead of the voting method. The learning method refers to the use of a single learner to learn and train the outputs of different individual learners and obtain the final result. By combining different individual learners with learning methods, the model generalization ability can be strengthened and the model bias can be reduced.

### 4.2 Subsequent research directions

1. So far, the deficiency of heart sound samples in patients with CHD-PAH greatly limits the development of aided diagnostic studies of CHD-PAH. Therefore, traditional machine learning was often used instead of deep learning in classification to prevent overfitting. However, compared with traditional machine learning, deep learning can learn features from input signals on its own, which is simpler and more convenient, but this depends on the background of big data.
2. At present, the signal acquisition work of our research group is still in progress to expand the self-constructed dataset. So more data will be used for algorithm optimization.
3. In the feature extraction phase of both algorithms, features were selected based on hyperacusis and splitting of the S2 component in patients with CHD-PAH. Additional features, such as pulmonary valve features, may be added to further enrich the features, according to clinical auscultation experience.
4. Current intelligent auscultation algorithms are based on specific datasets that have already been collected and selected. Therefore, in actual clinical screening, the performance of the algorithm needs to be validated.

### 4.3 Limitations of this review

1. Lack of comparison of the two algorithms: since the two algorithms use different datasets with different quality and quantity, it is inappropriate to directly compare the two algorithms by indicators such as classification accuracy.
2. Failure to analyze the most suitable algorithm in each stage of CHD-PAH recognition due to fewer related studies.

## 5 Conclusion

CHD-PAH is associated with a high clinical mortality. However, the current clinical diagnostic method—right cardiac catheterization is invasive and unsuitable for mass screening. And there are few academic studies on relevant classification algorithms. Therefore, two novel models for early non-invasive diagnosis of CHD-PAH, namely the direct three-divided model and two-stage classification model, were proposed by our research group.

For the direct three-divided model, a dual-threshold segmentation algorithm based on short-time energy and spectral spread was proposed in Ge et al. (2023) to segment the heart sound signal into cardiac cycles and S2. Based on them, time-domain features, frequency-domain features, energy features and deep features were extracted and combined into fusion features. In view of the small sample size, XGBoost was selected as the classifier. The majority voting algorithm synthesizes classification results across multiple cardiac cycles within a heart sound signal, achieving an accuracy of 88.61% on self-constructed dataset.

For the two-stage classification model, the experimental results show that it is effective to identify CHD-PAH on the basis of identifying pathological signals. The algorithm achieved an impressive accuracy of 90.9% under symmetric data and 93.3% under asymmetric data.

The above two models have a good performance in the aided diagnosis of CHD-PAH. It is hoped that it can be used in screening CHD-PAH. By considering the advantages and disadvantages of the two algorithms, future research directions of CHD-PAH assisted diagnosis were discussed. It is hoped that it will provide insight into prediction of CHD-PAH. Thus improving CHD-PAH predictive accuracy and reducing mortality.
